# Supplementary material for: Mitochondria-adaptor TRAK1 promotes kinesin-1 driven transport in crowded environments
Source: Nat Commun. 2020 Jun 19;11:3123. doi: 10.1038/s41467-020-16972-5 (PMC7305210; doi:10.1038/s41467-020-16972-5)
Supplement: Supplementary file 1 — Supplementary Information [file 41467_2020_16972_MOESM1_ESM.pdf]

Supplementary Information

Mitochondria-adaptor TRAK1 promotes kinesin-1 driven transport in crowded environments

Henrichs et al.

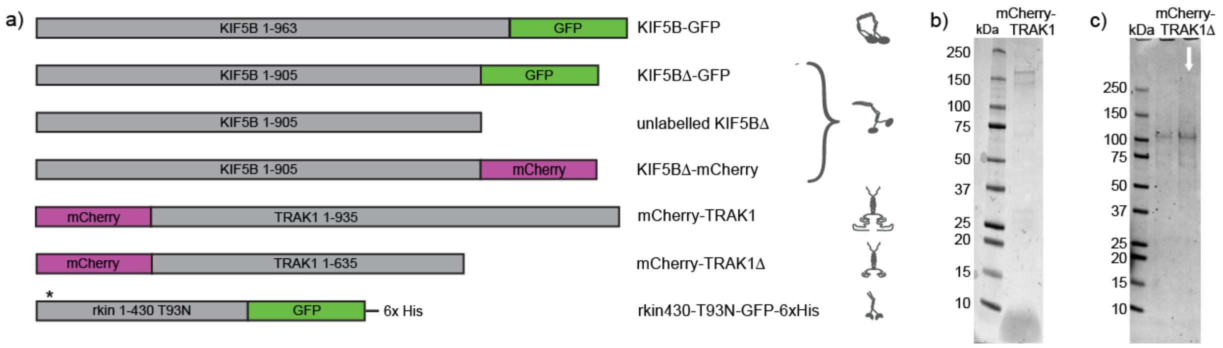

Supplementary Figure 1. Related to Figure 1-5. a) List of constructs used in this study. The asterisk indicates the position of the T93N point mutation of the rigor binding kinesin-1 mutant rkin430. **b)** SDS-gel of purified mCherry-TRAK1. **c)** SDS-gel of purified mCherry-TRAK1Δ. Source data are provided as a Source Data file.

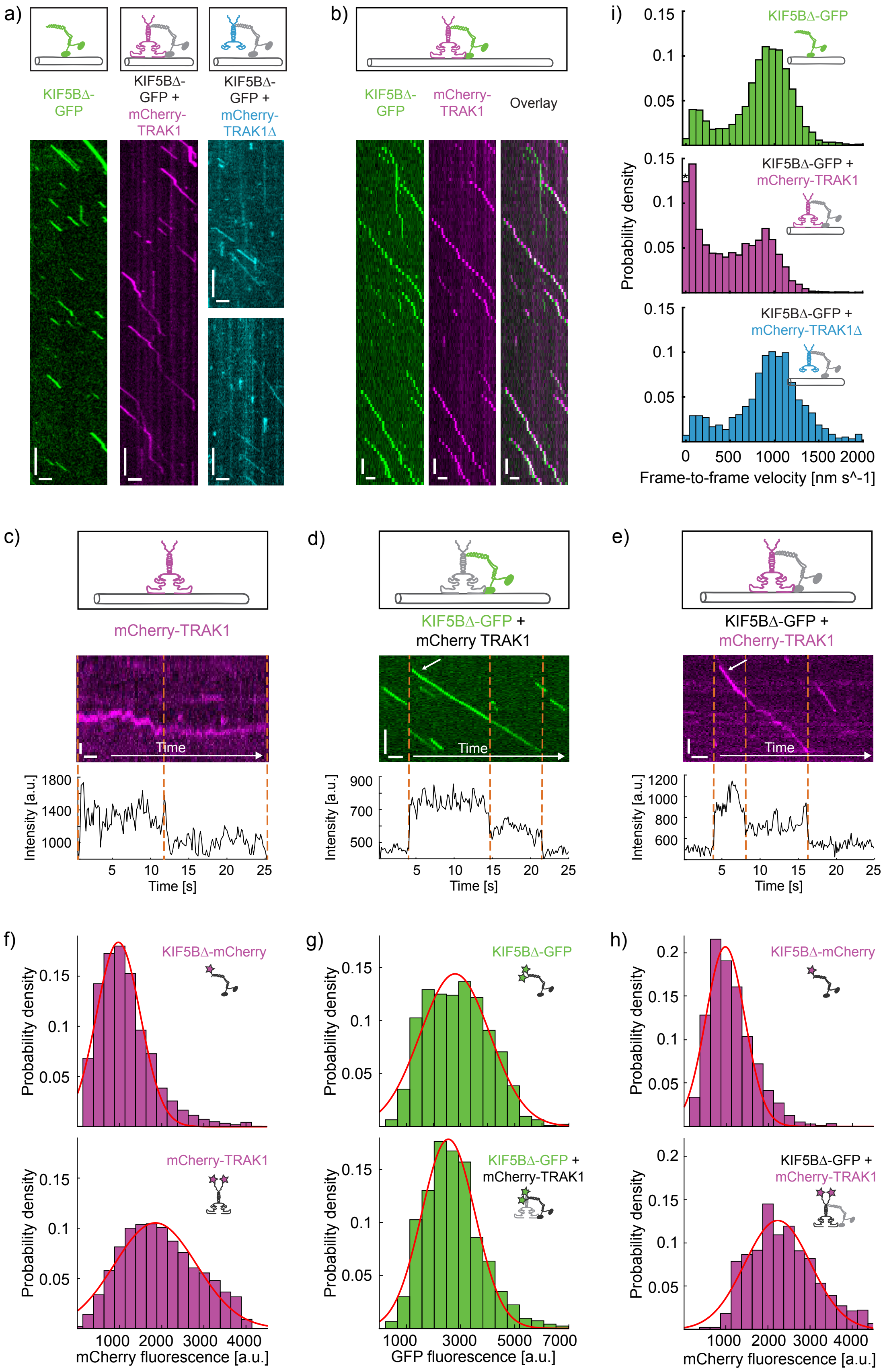

Supplementary Figure 2. Related to Figure 2 and 5. a) Schematic illustrations and kymographs of processively moving KIF5BΔ-GFP (green), mCherry-TRAK1 in presence of KIF5BΔ-GFP (magenta) and mCherry-TRAK1Δ in presence of KIF5BΔ-

GFP (cyan). Horizontal scale bars 2 µm, vertical 5 s. **b)** Schematic illustration and kymographs of KIF5BΔ-GFP colocalizing with mCherry-TRAK1 and walking processively along a microtubule. Horizontal scale bars 2 µm, vertical 5 s. **c) - e)** Schematic illustrations, kymographs and intensity time traces (Methods) showing two-step photobleaching of the mCherry signal of mCherry-TRAK1 diffusing along a microtubule and of the GFP and mCherry signal, respectively, of KIF5BΔ-GFP-mCherry-TRAK1 complexes walking processively along a microtubule, suggesting the presence of both molecules in a dimeric form. The white arrows indicate the molecules yielding the respective time traces. The dashed lines indicate the photobleaching steps. Horizontal scale bars 2 s, vertical 1 µm. **f)** Histograms of the background-subtracted integrated fluorescence intensities of the mCherry signal and Gaussian fits (red) of constitutively dimeric[31,77] KIF5BΔ-mCherry (top, *n* = 355 molecules) and of mCherry-TRAK1 (bottom, *n* = 256 molecules). Size exclusion chromatography measurements revealed that the large majority of KIF5BΔ-mCherry dimers harbor a single active mCherry fluorophore, while the large majority of mCherry-TRAK1 dimers harbor two active mCherry fluorophores (Methods, Supplementary Table 1). We thus interpret the single peak in the KIF5BΔ-mCherry intensity distribution as the signal of a single active mCherry fluorophore harbored by the (constitutively dimeric) KIF5BΔ and the single peak in the mCherry-TRAK1 distribution (centered at around twice the KIF5BΔ-mCherry intensity) as the signal of two active mCherry fluorophores harbored by a TRAK1 dimer. **g)** Histograms of the background-subtracted integrated fluorescence intensities of the GFP signal and Gaussian fits (red) of the constitutively dimeric KIF5BΔ-GFP (top, *n* = 79 molecules) and KIF5BΔ-GFP in complex with mCherry-TRAK1 (bottom, *n* = 150 molecules). The similar position of the peaks in both histograms indicates that a single KIF5BΔ-GFP dimer is present in a KIF5BΔ-GFP-mCherry-TRAK1 transport complex. **h)** Histograms of the background-subtracted integrated fluorescence intensities of the mCherry signal and Gaussian fits (red) of KIF5BΔ-mCherry (top, *n* = 33 molecules) harboring a single mCherry fluorophore per dimer (Methods, Supplementary Table 1) and mCherry-TRAK1 harboring two mCherry fluorophores per dimer (Methods, Supplementary Table 1) in complex with KIF5BΔ-GFP (bottom, *n* = 150 molecules). Analogously to f), the position of the peak in the histogram of mCherry-TRAK1 in complex with KIF5BΔ-GFP (centered at around twice the KIF5BΔ-mCherry intensity) indicates that a single TRAK1 dimer is present in a KIF5BΔ-GFP-mCherry-TRAK1 transport complex. **i) c)** Histograms of frame-to-frame

velocities of KIF5BΔ-GFP (green), KIF5BΔ-GFP in presence of mCherry-TRAK1 (magenta) and KIF5BΔ-GFP in presence of mCherry-TRAK1Δ (cyan). The median frame-to-frame velocity of KIF5BΔ-GFP decreased in presence of mCherry-TRAK1 from 909 nm s^-1 (lower, upper quantile limit 687 nm s^-1, 1085 nm s^-1, *n* = 534 molecules, *N* = 2 experiments) to 458 nm s^-1 (lower, upper quantile limit 97 nm s^-1, 842 nm s^-1, *n* = 487 molecules, *N* = 4 experiments). The population around zero (indicated by an asterisk) represents a high number of transient pauses. In presence of mCherry-TRAK1Δ the median frame-to-frame velocity of KIF5BΔ-GFP did not decrease (972 nm s^-1, lower, upper interquartile limit 732 nm s^-1, 1060 nm s^-1, *n* = 82 molecules, *N* = 2 experiments). Experiments were repeated at least 10 times with similar results. Source data are provided as a Source Data file.

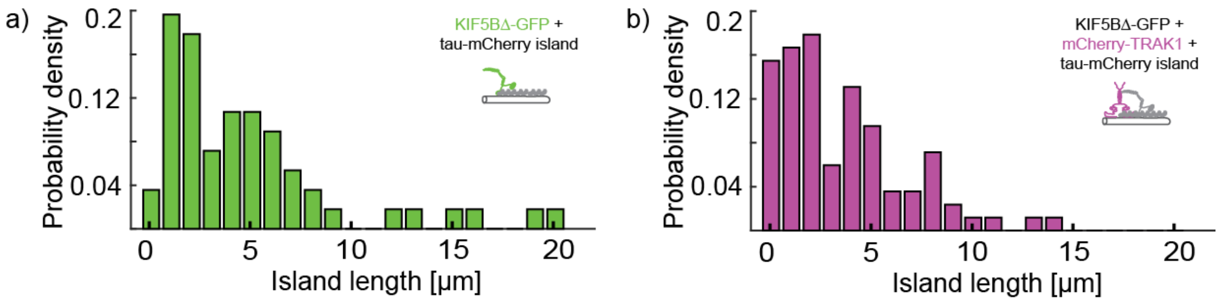

Supplementary Figure 3. Related to Figure 3. Histograms of the tau island length distribution in experiments with **a)** KIF5BΔ-GFP (4.75 ± 4.8 μm, mean ± standard deviation, n = 56 islands, N = 4 experiments) and **b)** KIF5BΔ-GFP-mCherry-TRAK1 complexes (3.54 ± 3.12 μm, mean ± standard deviation, n = 84 islands, N = 4 experiments). Source data are provided as a Source Data file.

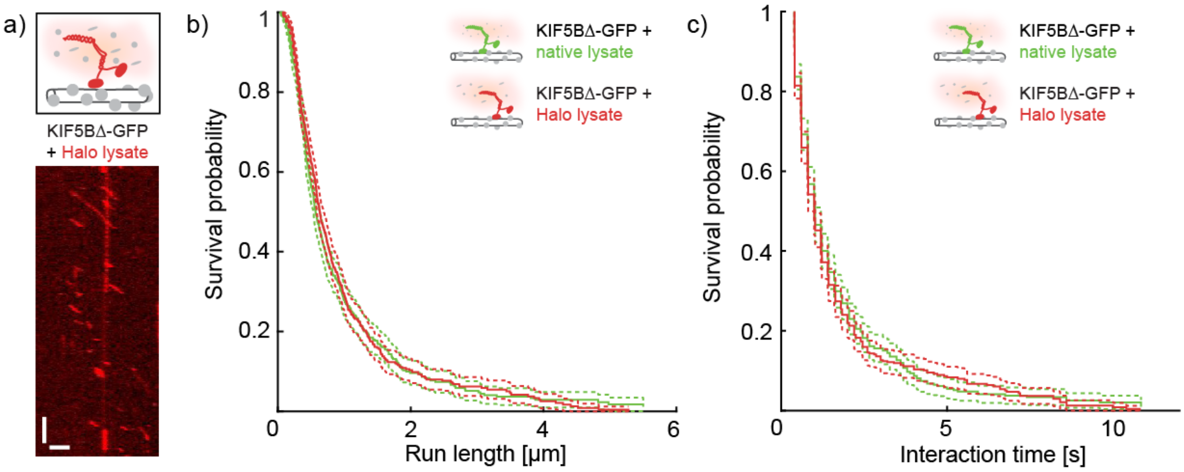

Supplementary Figure 4. Related to Figure 4. a) Schematic illustration and kymograph of processively moving KIF5BΔ-GFP in Halo lysate. Horizontal scale bar 1 µm, vertical 10 s. **b), c)** Survival probabilities indicating a similar median run length (0.63 µm (CI_{95} (0.58, 0.69) µm, *n* = 555 molecules, *N* = 6 experiments)) and interaction time (1 s (CI_{95} (0.82, 1.02) s, *n* = 555 molecules, *N* = 6 experiments)) of KIF5BΔ-GFP in Halo lysate (red) in comparison to native lysate (green) (compare to Figure 4h,i) (*p* = 0.6 and *p* = 0.3, respectively). Two-tailed *p*-values were obtained by a log-rank test. Source data are provided as a Source Data file.

a)

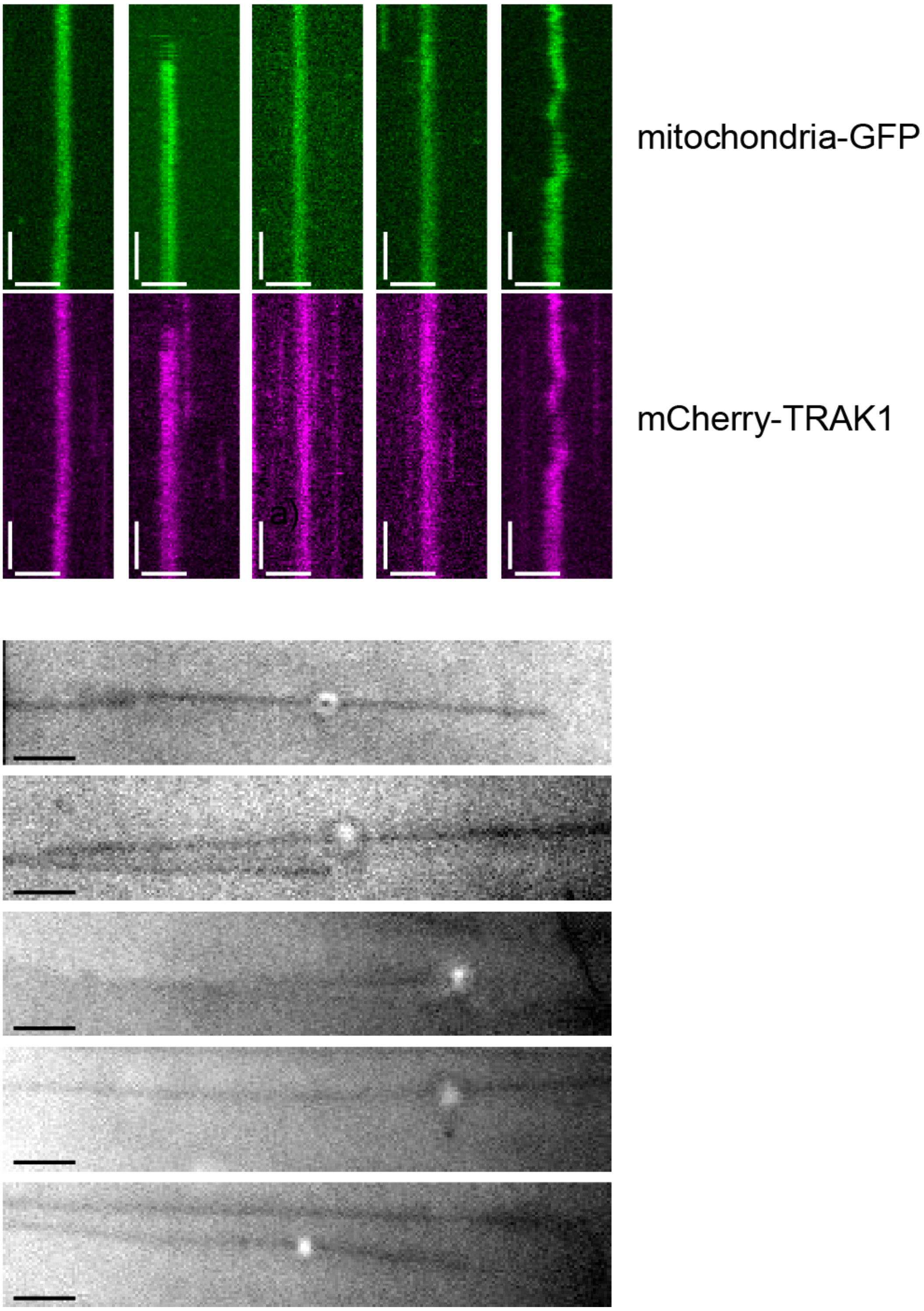

b)

Supplementary Figure 5. Related to Figure 5. a) Kymographs showing stationary and diffusive tethering of mitochondria-GFP (green) to microtubules by mCherry-TRAK1 (magenta). The experiment was repeated 5 times independently with similar results). Horizontal scale bars 2 µm, vertical 10 s. **b)** Representative interference reflection microscopy images of single mitochondria bound to microtubules. Scale bars 2 µm.

Supplementary Table 1: Values for calculating the labelling efficiencies of KIF5BΔ-mCherry and mCherry-TRAK1

	**KIF5BΔ-GFP**	**mCherry-TRAK1**
Absorbance at 280 nm [AU] (A_{280})	35.96	44.00
Absorbance at 561 nm [AU] (A_{561})	6.85	33.95
Extinction coeff. of protein at 280 nm [M^-1 cm^-1] ($\mathcal{E}_{280}$)	82500	82000
Extinction coeff. of protein at 561 nm [M^-1 cm^-1] ($\mathcal{E}_{561}$)	72000	72000
Labelling efficiency	0.2183	0.8787

A is the absorbance and $\mathcal{E}$ the extinction coefficient at 280 nm (A_{280} and $\mathcal{E}_{280}$) and 561 nm (A_{561} and $\mathcal{E}_{561}$), respectively.

Supplementary Table 2: Primers used in this work.

	Forward primers	Reverse primer	PCR
**mCherry-TRAK1**	TCGGAGAACCTGTACTTCCAGTCTACCATGGTGAGCAAGGGCGAGGAGGATAACATG	GGGGACCACTTTGTACAAGAAAGCTGGGTTATTACCGTAAGCTAGTTTGTTTGGAGAG	Gateway step 1
**mCherry-TRAK1**	GGGGACAAGTTTGTACAAAAAAGCAGGCTCGGAGAACCTGTACTTCCAG	GGGGACCACTTTGTACAAGAAAGCTGGGTTATTACCGTAAGCTAGTTTGTTTGGAGAG	Gateway step 2
**mCherry-TRAK1Δ**	GACACAGGTGACCACTAAATTTCTCTCCCACGC	GCGTGGGAGAGAAATTTAGTGGTCACCTGTGTC	Mutagenesis
**KIF5B**	AATAATAACATGCGGCCGCAATGGCGGACCTGGCCGAGTG	AATAATAACATGGCGCGCCCACTTGTTTGCCTCCTCCACCTC	Amplification
**KIF5BΔ**	AATAATAACATGCGGCCGCAATGGCGGACCTGGCCGAGTG	AATAATAACATGGCGCGCCTGCTTCCTTTATGCGATCTACTTCTTGC	Amplification
