# Supplementary material for: Mitochondria-adaptor TRAK1 promotes kinesin-1 driven transport in crowded environments
Source: Nat Commun. 2020 Jun 19;11:3123. doi: 10.1038/s41467-020-16972-5 (PMC7305210; doi:10.1038/s41467-020-16972-5)
Supplement: Supplementary file 3 — Description of Additional Supplementary Files [file 41467_2020_16972_MOESM3_ESM.pdf]

Description of Additional Supplementary Files

File Name: Supplementary Data 1

Description: Mass Spectrometry on three individual samples of mitochondria verified that these mitochondria did not contain the adaptor protein TRAK1 but the trans-membrane protein Miro which binds mitochondria to the KIF5B-TRAK1 transport complex.
